# Betaine Attenuates Osteoarthritis by Inhibiting Osteoclastogenesis and Angiogenesis in Subchondral Bone

**DOI:** 10.3389/fphar.2021.723988

**Published:** 2021-09-29

**Authors:** Wang Yajun, Cui Jin, Gu Zhengrong, Fang Chao, Hu Yan, Weng Weizong, Li Xiaoqun, Zhou Qirong, Chen Huiwen, Zhang Hao, Guo Jiawei, Zhuang Xinchen, Sheng Shihao, Wang Sicheng, Chen Xiao, Su Jiacan

**Affiliations:** ^1^ Graduate Management Unit, Shanghai Changhai Hospital, Naval Medical University, Shanghai, China; ^2^ Department of Orthopedics, Shanghai Changhai Hospital, Naval Medical University, Shanghai, China; ^3^ Department of Orthopedics, Luodian Hospital, Shanghai, China; ^4^ Institute of Translational Medicine, Shanghai University, Shanghai, China; ^5^ Department of Orthopedics, Zhongye Hospital, Shanghai, China; ^6^ Shanghai Clinical Research Center for Aging and Medicine, Shanghai, China

**Keywords:** betaine, osteoclastogenesis, subchondral bone, angiogenesis, osteoarthritis

## Abstract

Osteoarthritis (OA) is the most common type of arthritis with no effective therapy. Subchondral bone and overlying articular cartilage are closely associated and function as “osteo-chondral unit” in the joint. Abnormal mechanical load leads to activated osteoclast activity and increased bone resorption in the subchondral bone, which is implicated in the onset of OA pathogenesis. Thus, inhibiting subchondral bone osteoclast activation could prevent OA onset. Betaine, isolated from the Lycii Radicis Cortex (LRC), has been demonstrated to exert anti-inflammatory, antifibrotic and antiangiogenic properties. Here, we evaluated the effects of betaine on anterior cruciate ligament transection (ACLT)-induced OA mice. We observed that betaine decreased the number of matrix metalloproteinase 13 (MMP-13)-positive and collagen X (Col X)-positive cells, prevented articular cartilage proteoglycan loss and lowered the OARSI score. Betaine decreased the thickness of calcified cartilage and increased the expression level of lubricin. Moreover, betaine normalized uncoupled subchondral bone remodeling as defined by lowered trabecular pattern factor (Tb.pf) and increased subchondral bone plate thickness (SBP). Additionally, aberrant angiogenesis in subchondral bone was blunted by betaine treatment. Mechanistically, we demonstrated that betaine suppressed osteoclastogenesis *in vitro* by inhibiting reactive oxygen species (ROS) production and subsequent mitogen-activated protein kinase (MAPK) signaling. These data demonstrated that betaine attenuated OA progression by inhibiting hyperactivated osteoclastogenesis and maintaining microarchitecture in subchondral bone.

## Introduction

Osteoarthritis (OA), which affects nearly 22% of those aged 60 years and older (Brooks, 2002; Gore et al., 2011), is the most common type of arthritis characterized by progressive degenerative joint disorder. The pathogenesis of OA is considered multifactorial, such as mechanical, metabolic or genetic components, and results in the characteristic phenotype of OA, including cartilage deterioration, subchondral bone edema and sclerosis, abnormal angiogenesis and osteophyte formation (Goldring and Goldring, 2016; Robinson et al., 2016). Although a number of pharmacological management strategies, including nonsteroidal anti-inflammatory drugs (NSAIDs), corticosteroids, protease inhibitors, stem cell therapies and nonpharmacological approaches such as physical activity, weight loss, and joint replacement, have been employed for OA, effective disease-modifying treatment is still lacking (Le Graverand-Gastineau, 2010; Fernandopulle et al., 2017; Latourte et al., 2020).

Subchondral bone remodeling plays a crucial role during OA development, especially in the early stage (Pan et al., 2009; Roemer et al., 2009; Tanamas et al., 2010). Subchondral bone acts as a mechanical girder and energy absorber and supports superficial articular cartilage during joint movement (Lories and Luyten, 2011; Saltzman and Riboh, 2018). Changes in the microarchitecture of subchondral bone affect the overlying joint cartilage and precede articular cartilage degradation in OA (Lajeunesse and Reboul, 2003; Kothari et al., 2010; Loeser et al., 2012). Subchondral bone microarchitecture was maintained by normal osteoclast activity and coupled bone remodeling. Osteoclast activity is significantly increased after injury, such as anterior cruciate ligament tears, leading to a substantial increase in bone resorption and uncoupled bone remodeling, which ultimately results in OA onset (Hayami et al., 2012; Khorasani et al., 2015). Inhibition of hyperactivated osteoclast activity in subchondral bone could ameliorate osteoarthritic severity (Siebelt et al., 2014; Geurts et al., 2016). In addition, abnormal angiogenesis facilitated by bone resorption promotes the diffusion of inflammatory and procatabolic mediators into the superficial articular cartilage, contributing to cartilage damage in the development of OA (Suri et al., 2007; Mapp et al., 2010).

Betaine, which was screened by a two-dimensional bone marrow mononuclear cell membrane chromatography system (2D BMMC/CMC system) from the Lycii Radicis Cortex (LRC), has been proven to possess multiple pharmacological characteristics, including antidiabetic, antioxidant and anti-inflammatory effects, by inhibiting nuclear factor kappa-B (NF-κB) and MAPK signaling (Yang et al., 2018; Shi et al., 2019; Veskovic et al., 2019; Dibaba et al., 2020). It was also reported that betaine could inhibit aberrant angiogenesis through suppression of the AKT, ROS and ERK pathways (Kim et al., 2015; Park et al., 2017). There is limited knowledge about the effects of betaine on OA.

In this study, we found that betaine attenuated OA progression by inhibiting hyperactivated osteoclastogenesis and maintaining microarchitecture in subchondral bone. Mechanistically, betaine suppresses osteoclastogenesis by reducing ROS production and inhibiting MAPK signaling.

## Materials and Methods

### Active Constituent Identification

A two-dimensional bone marrow mononuclear cell membrane/chromatography/C18 column/time-of-flight mass spectrometry assay was performed to screen active components of affinity. Briefly, cell membrane chromatography (CMC) was utilized to screen active components in the LRC. TOFMS was used to detect and trace components. Ingredients with long retention times and better retention behavior were regarded as potential active constituents. Bone marrow monocyte cells (BMMCs) isolated from the femoral cavity of mice were cultured with LRC, and then the retention time of each component was screened by an Agilent MassHunter Workstation (Agilent Technologies, Palo Alto, CA, United States).

### Cell Viability Assay

A CCK-8 (R&D Systems, Minneapolis, MN, United States) assay was adopted to detect betaine (RHAWN, R20086) cytotoxicity following the manufacturer’s protocol. In brief, BMMCs at a concentration of 10 × 10^4^ cells/ml were added to 96-well plates. After 24 h, multiple concentrations of betaine (0, 31.25, 62.5, 125, 250, 500, 1,000 , 2000 μM) were added to the wells and incubated for another 48 h. Then, CCK-8 solution (10 μl) was added and cocultured for 2 h in an incubator. The absorbance level of each well at 450 nm was determined by an ELISA microplate reader.

### Osteoclastogenesis Assay

BMMCs isolated from 4-week-old C57BL/6 mice were cultured in α-MEM supplemented with FBS (HyClone, Logan, UT, United States) and an antibiotic mixture (HyClone, Logan, UT, United States) on a petri dish at 37°C and 5% CO_2_. After 72 h, nonadherent cells were extracted, and the remaining cells were subcultured. Third-generation BMMCs were divided into a control group and groups treated with different concentrations of betaine. BMMCs of the betaine-treated groups were induced to differentiate into osteoclasts by M-CSF (20 ng/ml) and RANKL (50 ng/ml) (R&D Systems, Minneapolis, MN, United States) with incremental concentrations of betaine (0, 125, 250, 500 μM). After 5 d, cells were stained with a TRAP staining kit (Sigma-Aldrich, St. Louis, MO, United States) following the manufacturer’s protocol. RAW264.7 cells were treated similarly as described above and stained with a TRAP staining kit after 5 d.

### Immunofluorescence Staining

Immunofluorescence was conducted to evaluate whether betaine treatment affects actin ring formation. BMMCs (1 × 10^4^ cells/well) were seeded on 96-well plates, stimulated with M-CSF (20 ng/ml) and RANKL (50 ng/ml) and cultured with or without betaine (500 μM) for 2 days. After the cells were fixed with PFA and permeabilized with Triton-X, an anti-F-actin antibody and a secondary antibody were added. Images were obtained by using an Olympus BX53 microscope.

### Bone Resorption Assay

A bone resorption assay was applied to examine the effect of betaine treatment on osteoclast function. After BMMCs were induced to mature osteoclasts, cells were digested and seeded on bone biomimetic synthetic surfaces (1 × 10^4^ cells/well) (Corning, Lowell, MA, United States) with M-CSF (20 ng/ml) and RANKL (50 ng/ml). Cells were incubated with or without betaine (500 μM) for an additional 2 days. On day 3, the surfaces were washed with PBS and air dried for 5 h. The pit area was counted and analyzed using an Olympus IX74 microscope and ImageJ software (NIH, Bethesda, MD, United States).

### Animals

All experimental protocols and feeding and maintenance of mice were performed in compliance with the rules of the Ethics Committee of Changhai Hospital (SYXK 2015–0017). Eight-week-old male C57BL/6 mice obtained from Weitonglihua Corporation (Beijing, China) were separated into three groups: sham-operated group, vehicle-treated ACLT group and betaine-treated ACLT group. For the ACLT group, we transected the anterior cruciate ligament of the right knee joint with microscissors to establish an abnormal mechanical loading-induced OA model. For the sham group, the skin and capsule of the right knee joint were opened under a microscope, and then the incision was sutured without transecting the anterior cruciate ligament. Beginning the second day after the operation, betaine at 2% w/v was dissolved in daily drinking water. Mice were euthanized at 14, 30, and 60 days after surgery separately. All experiments were performed under pathogen-free conditions.

### Histochemistry, Immunohistochemistry and Histomorphometry

Right knee joint specimens were taken at 14, 30, and 60 days after surgery, fixed in 10% formalin for another 2 d and decalcified in 10% EDTA (pH 7.3) (Solarbio, Beijing, China) for 14 d. Longitudinal-oriented sections (4-μm-thick) of the medial compartment of the right knee joint were cut and prepared for safranin O and fast green staining, TRAP staining and HE staining. The connective line between hyaline cartilage and calcified cartilage was determined as the tidemark line. The length from the articular cartilage surface to the tidemark line was determined to be hyaline cartilage, and the length from the tidemark line to the subchondral bone plate was regarded as calcified cartilage. Sagittal-oriented sections of the specimen were prepared at 4°C. Then, primary antibodies against collagen X (Abcam, 1: 50, ab260040), MMP13 (Abcam, 1: 400, ab219620), lubricin (Abcam, 1: 50, ab28484), TRAP (Abcam, 1:40, ab133238), CD31 (Abcam, 1:100, ab28364), MMP-2 (Abcam, 1:1,000, ab97779), and endomucin (Abcam, 1:100, ab106100) were incubated overnight. For immunofluorescence staining, we incubated secondary antibodies for 1 h while avoiding light at room temperature. For histomorphometric measurement, the tibial subchondral bone area was determined by using an Olympus BX53 microscope. The histologic score of OA, which was measured by the Osteoarthritis Research Society International-modified (OARSI) Mankin criteria score, was quantified as described (Glasson et al., 2010).

### Micro-CT Analysis

The knee joint without soft tissue was scanned by micro-CT (SkyScan 1,176, United States) at a resolution of 9 μm per pixel, 80 kVp voltage and 124 μA current after fixation in formalin overnight. We defined the region of interest by using Data-Viewer software (Version 1.5) to cover the entire area of the medial compartment of the tibia subchondral bone. The data were then analyzed by CTAn (Version 1.8), and a three-dimensional model was visualized by CTVol (Version 2.0). The structural parameters were analyzed as follows: BV/TV (bone volume/tissue volume), SBP Th (subchondral bone plate thickness) and Tb. Pf (trabecular pattern factor).

### Microangiography

The thoracic cavity and right atrium of mice were opened by scissors, and a syringe needle was punctured into the left ventriculus sinister after anesthetization. The blood vessels were flushed with 0.9% normal saline with 100 μ/ml heparin sodium added through the needle. After that, the mice were flushed and fixed with formalin through the syringe and washed with heparinized 0.9% normal saline. Then, we injected radiopaque silicone rubber compound (Microfil MV-120, Flow Tech) containing lead chromate into the vascular system. Mice were preserved at 4°C for 24 h. The knee joint specimens were dissected and harvested and soaked in formalin for another 2 d. Specimens were decalcified in EDTA for 14 days to lessen the effect of surrounding bone tissues. Micro-CT (SkyScan 1,176, United States) at 40 kVp voltage, 80 μA current and 10 μm resolution per pixel was used to acquire images.

### Tube Formation Assay

Human umbilical vein endothelial cells (HUVECs) (2 × 10^4^ cells/well) were seeded on previously polymerized Matrigel (Corning, NY, United States) in 96-well plates and cultured with or without betaine (500 μM) at 37°C and 5% CO_2_. After 12 h, morphologic changes of cells were observed and photographed at ×40 magnification.

### Wounding Migration Assay

HUVECs were seeded onto 24-well plates and cultured to confluence. After overnight starvation with basal medium, cells were wounded with a 200 μl pipette tip. Then, the cells were washed with PBS three times and incubated with serum-free medium with or without betaine (500 μM). After 24 h, migration was photographed and quantitated by calculating the scratch width between the reference line.

### PCR Analysis

Whole RNA was extracted from HUVECs with TRIzol reagent (Invitrogen, Carlsbad, CA, United States). The following PCR primers were used: bFGF forward (5′-TGC TGT TTC TAT GTC GTG GAA-3′), bFGF reverse (5′-AGG CAG TGC TGA TTT TCA GTC-3′), MMP-2 forward (5′-TAC AAC TTC TTC CCT CGC AAG-3′), MMP-2 reverse (5′-AAA GGC ATC ATC CAC TGT CTC-3′), MMP-9 forward (5′-GCT GGG CTT AGA TCA TTC CTC-3′), MMP-9 reverse (5′-ATT CAC GTC GTC CTT ATG CAA-3′).

### Reactive Oxygen Species Production Assay

ROS (Beyotime, S0033S) assays were performed to detect the intracellular ROS level according to the instructions. Briefly, BMMCs were pretreated with or without betaine (500 μM), M-CSF (20 ng/ml) and RANKL (50 ng/ml) for 24 h. After that, the cells were incubated with an ROS probe (1:1,000) for 20 min. The fluorescence intensity of DCF and ROS-positive cells was counted and analyzed using an Olympus IX74 microscope and ImageJ software (NIH, Bethesda, MD, United States).

### Western Blotting

Western blot analysis was performed to analyze the influence of betaine treatment on the MAPK pathway, ROS-related enzymes and osteoclastogenesis-related markers. We used M-PER mammalian protein extraction reagent (Pierce, Rockford, IL, United States) to extract total protein from BMMCs. Twenty micrograms of protein sample per lane was loaded onto (11%) SDS-PAGE gels. When the protein sample reached the bottom of the gel, the power was cut off, electrophoresis was stopped, and the protein bands were transferred to polyvinylidene fluoride (PVDF) membranes (Bio-Rad, 1620177). The primary antibodies included anti-P38 (Abcam, 1:1,000, ab170099), anti-P-P38 (Abcam, 1:1,000, ab195049), anti-ERK (Abcam, 1:1,000, ab17942), anti-P-ERK (Abcam, 1:1,000, ab201025), anti-JNK (Abcam, 1:1,000, ab179461), anti-P-JNK (Abcam, 1:1,000, ab124956), anti-TRAF6 (Abcam, 1:1,000, ab137452), anti-Nox1 (Abcam, 1:1,000, ab121009), anti-HO1 (Abcam, 1:1,000, ab137749), anti-catalase (Abcam, 1:1,000, ab217793), anti-TRAP (Abcam, 1:5,000, ab52750), anti-MMP9 (Abcam, 1:1,000, ab228402), and anti-Cathepsin K (Abcam, 1:1,000, ab187647), followed by the secondary antibody (Biosharp, 1:5,000, BL003A). The results were probed by chemiluminescence.

### Statistical Analysis

The results are expressed as the mean ± standard deviation (SD). GraphPad Prism (version 6, GraphPad Software, San Diego, CA, United States) was applied to analyze the data. Differences between two groups were analyzed by Student’s t-test, while variance between three or more groups was compared by one-way ANOVA. *p* < 0.05 was indicated to be significant.

## Results

### Betaine Suppresses Osteoclastogenesis *in vitro*

**FIGURE 1:**
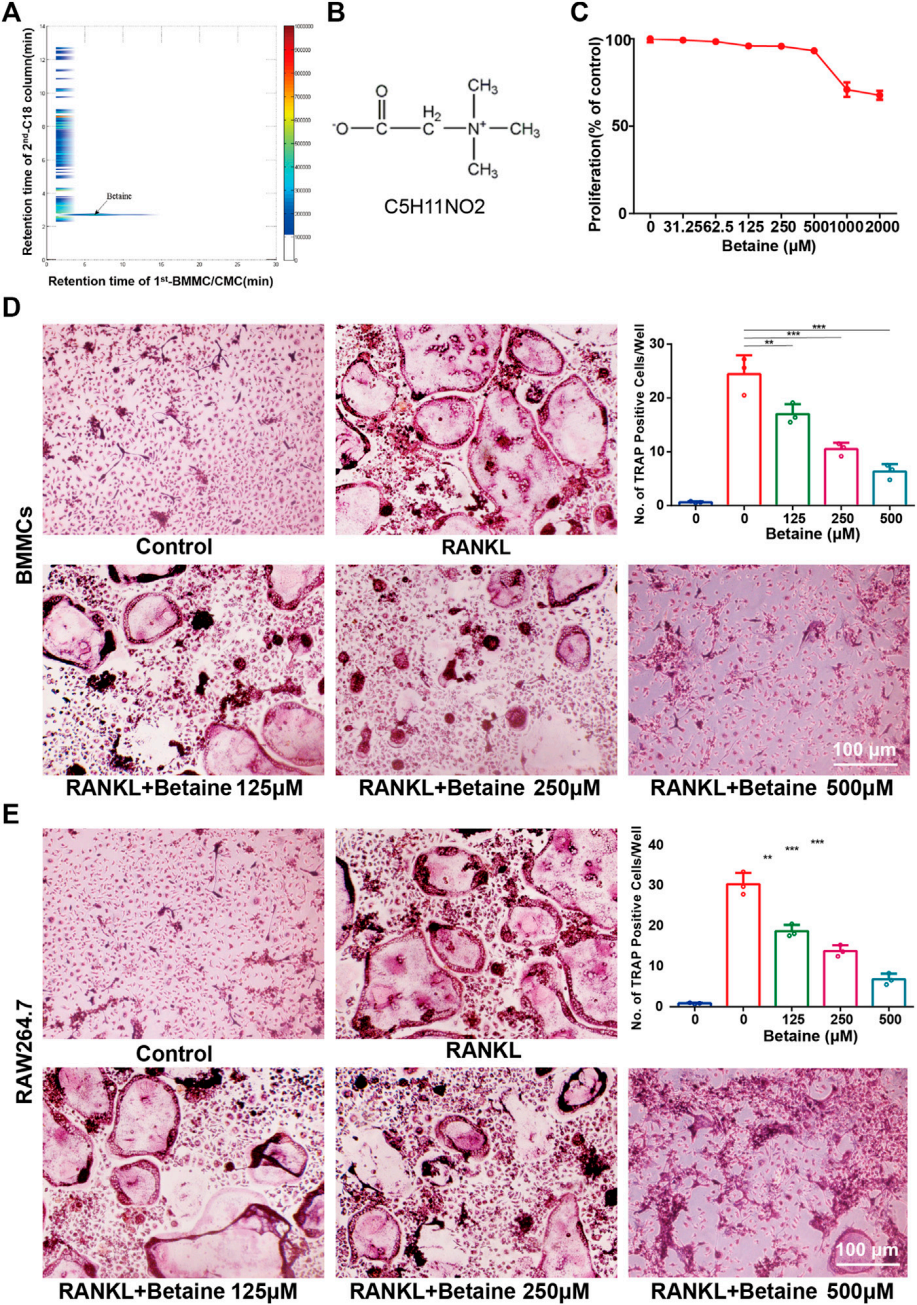
Betaine suppresses osteoclastogenesis *in vitro*. **(A)** Typical 2D plots of Lycii Radicis Cortex (LRC) by 2D BMMC/CMC/C18 column/TOFMS assay system. **(B)** Chemical formula of betaine. **(C)** CCK-8 assay of betaine’s cytotoxicity in BMMCs. **(D)** Formation and quantification of tartrate-resistant acid phosphatase (TRAP) positive osteoclast cells from BMMCs. **(E)** Formation and quantification of TRAP positive osteoclast cells from RAW264.7 cells. *p < 0.05 as denoted by bar, **p < 0.01 as denoted by bar, ***p < 0.001 as denoted by bar.

**FIGURE 2:**
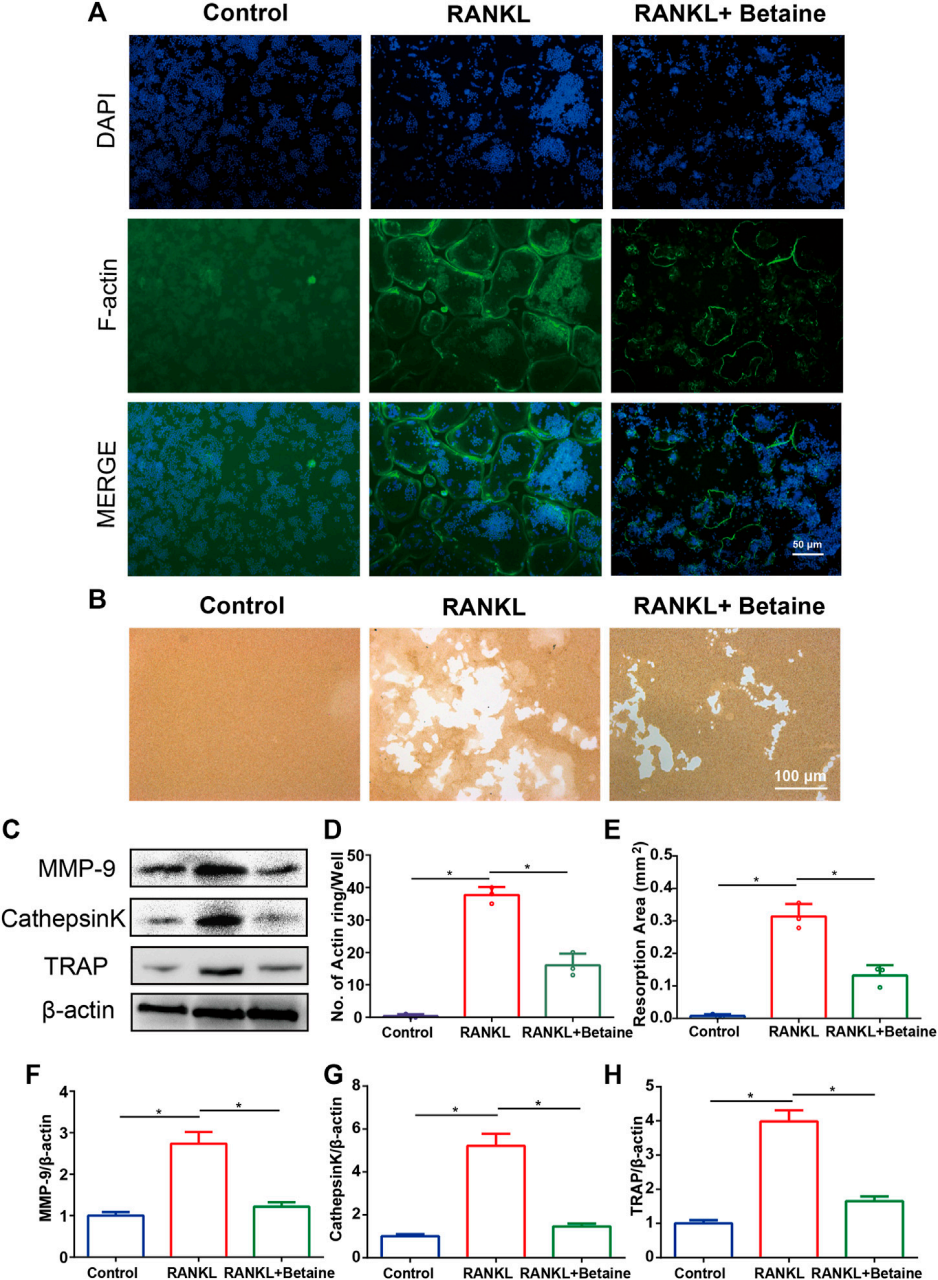
Betaine inhibits osteoclasts function *in vitro*. **(A)** F-actin ring formation of osteoclasts. Scale bar, 50 μm. **(B)** Pit form assay of osteoclasts. Scale bar, 100 μm. **(C)** Western blot of the expression of MMP-9, CathepsinK and TRAP. **(D)** Quantification of F-actin ring formation. **(E)** Quantification of pits area. **(F–H)** Quantification of MMP-9, CathepsinK and TRAP in different groups. *p < 0.05 as denoted by bar, **p < 0.01 as denoted by bar, ***p < 0.001 as denoted by bar.

2D CMC analysis was applied, and possible active constituents were screened from LRC using bone marrow monocyte cells (BMMCs). Betaine showed better retention behavior on BMMCs, indicating a better biological affinity to receptors on BMMC membranes (Figures 1A,B). This component was expected to play a role in inhibiting osteoclastogenesis. CCK-8 assay following the manufacturer’s protocol was performed to detect betaine cytotoxicity. The results showed that betaine had no cytotoxicity below 500 μM (Figure 1C). BMMCs and RAW264.7 cells were used to explore the potential impact of betaine on osteoclastogenesis. The results indicated that the number of TRAP^+^ osteoclasts was dramatically increased after induction by M-CSF and RANKL, and betaine at 125, 250, and 500 μM significantly decreased the number of TRAP^+^ osteoclasts (Figures 1D,E). Furthermore, actin ring formation and pit formation assays were adopted to examine the effects of betaine on osteoclast bone resorption function. Betaine treatment significantly reduced the number of actin rings, which are prerequisites for bone resorption (Figures 2A,D). In parallel, obvious pits were formed in the plates after BMMCs were induced by RANKL and M-SCF, and betaine treatment decreased the resorbed area (Figures 2B,E). Additionally, western blot analysis showed that the expression of osteoclastogenesis-related markers, including Cathepsin K, MMP 9 and TRAP, was significantly inhibited by betaine treatment (Figures 2C,F–H). These results suggest that betaine could inhibit osteoclastogenesis and osteoclast bone resorption function *in vitro*.

### Betaine Attenuates Cartilage Degeneration in OA Mice

**FIGURE 3:**
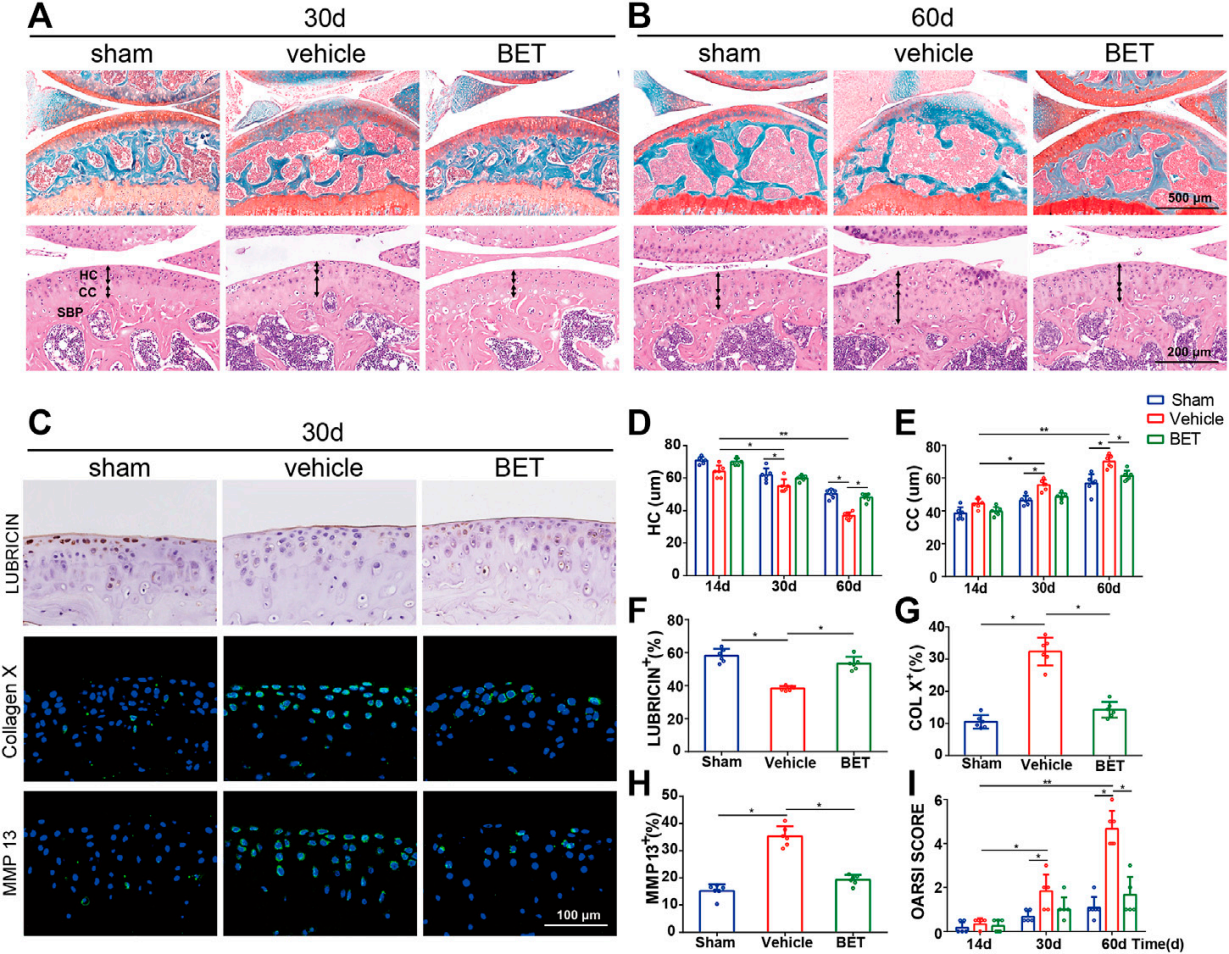
Betaine attenuates cartilage degeneration in OA mice. **(A,B)** Safranin O and fast green staining (top) showed proteoglycan loss and cartilage damage 30 and 60 days after operation. Scale bar, 500 μm. Double-headed arrows marked hyaline cartilage (HC) and calcified cartilage (CC) thickness in H&E staining (bottom). Scale bars, 200 μm. **(C)** Immunostaining of lubricin (upper), COL X (middle) and matrix metalloproteinase (MMP) 13 (lower) in articular cartilage of keen joint 30 days after surgery. Scale bar, 100 μm. **(D,E)** Changes of the HC and CC thicknesses in each group and time point. **(F–H)** Quantitative analysis of lubricin, COL X and MMP 13 expression in cartilage 30 days after surgery. **(I)** OA Score of articular cartilage degradation in each group and time point. *p < 0.05 as denoted by bar, **p < 0.01 as denoted by bar.

We investigated the effects of betaine on ACLT-induced OA progression. Safranin O and fast green staining results showed that articular cartilage proteoglycan was significantly reduced in vehicle-treated ACLT group mice at 30 and 60 d after operation. Betaine treatment (2% w/v) significantly preserved proteoglycan loss in ACLT mice (Figures 3A,B). This result was also supported by OARSI scores indicating that no difference was noted between betaine-treated mice and sham controls, which were significantly improved compared with vehicle-treated mice (Figure 3I). HE staining showed that calcified cartilage thickness was significantly increased and hyaline cartilage thickness was considerably decreased in vehicle-treated mice at 30 and 60 d, both of which were normalized by betaine administration (Figures 3A,B,D,E). In addition, immunostaining results suggested that betaine administration improved the expression level of lubricin, which was substantially reduced in vehicle-treated groups compared with sham controls at 30 d (Figures 3C,F). Conversely, the increased levels of MMP-13 and collagen X in articular cartilage of vehicle-treated groups relative to sham controls at 30 d were significantly decreased by betaine administration (Figures 3C,G,H). These results suggest that betaine could protect articular cartilage from deterioration in ACLT mice.

### Betaine Maintains Subchondral Bone Microarchitecture in OA Mice

**FIGURE 4:**
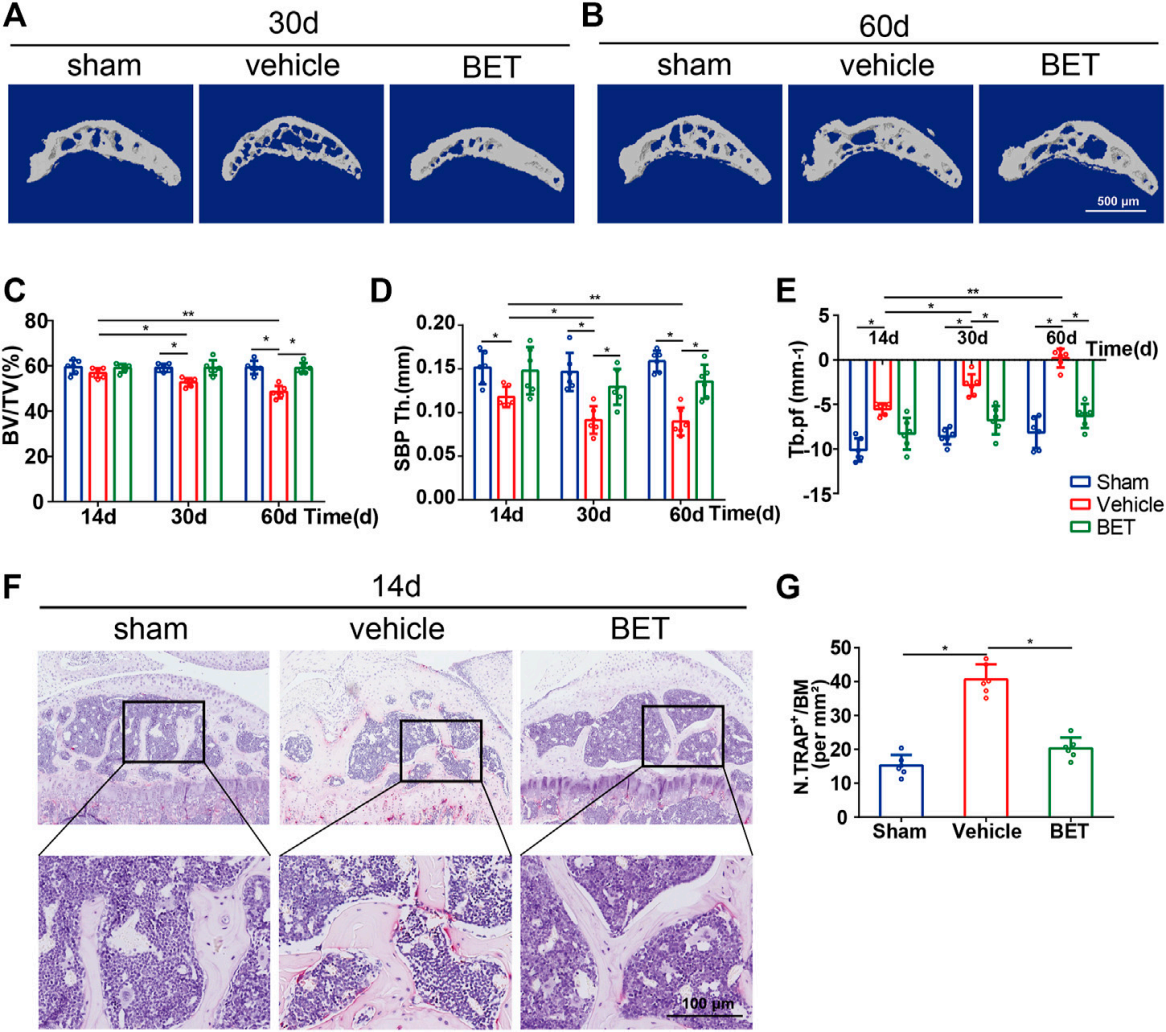
Betaine maintains subchondral bone microarchitecture in ACLT mice. **(A,B)** Sagittal 3D micro-CT images of subchondral bone medial compartment 30- and 60-days post operation. Scale bar, 500 μm. **(C–E)** Quantitative analysis of bone volume/tissue volume (BV/TV) of tibial subchondral bone, subchondral bone plate thickness and trabecular pattern factor (Tb.pf). **(F)** TRAP staining in subchondral bone 14 days after operation. Scale bar, 100 μm. **(G)** Quantification analysis of TRAP staining in subchondral bone 14 days after operation. *p < 0.05 as denoted by bar, **p < 0.01 as denoted by bar.

Micro-CT was applied to analyze whether betaine supplementation had a possible effect on the microstructure of tibial subchondral bone. The results suggested that the value of BV/TV was significantly decreased in ACLT mice at 30 and 60 d. Betaine maintained it to a level comparable to sham controls (Figures 4A–C). Similarly, Tb. pf (a parameter of bone resorption) was significantly increased in the vehicle-treated groups at 30 and 60 d and was decreased by betaine (Figure 4E). Conversely, SBP Th (a parameter of bone formation) was substantially reduced at 30 and 60 d post operation, while betaine administration increased it to a similar level as the sham controls (Figure 4D). In addition, immunostaining results demonstrated that betaine significantly reduced TRAP^+^ osteoclast numbers in subchondral bone marrow at 14 d, whereas no difference was observed between betaine-treated mice and sham controls (Figures 4F,G). These data suggest that betaine could maintain subchondral bone microarchitecture by inhibiting hyperactivated osteoclastogenesis and inhibiting increased bone resorption in ACLT mice.

### Betaine Suppresses Blood Vessel Formation *in vitro*

**FIGURE 5:**
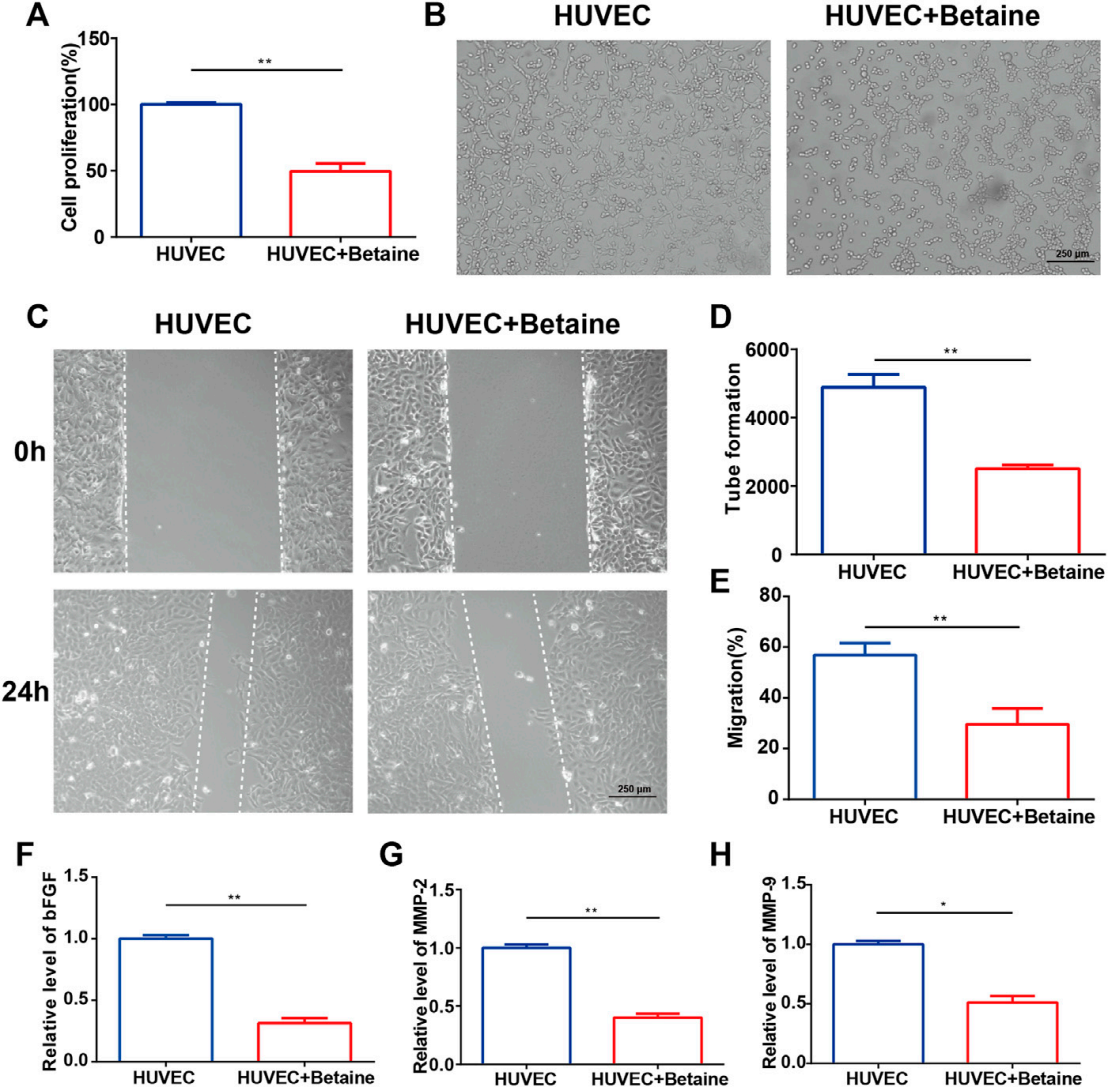
Betaine suppresses blood vessel formation *in vitro*. **(A)** Proliferation of HUVECs in different groups. **(B)** Tube formation assay of HUVECs for 12 h. Scale bar, 250 μm. **(C)** Wounding migration assay of HUVECs for 24 h. Scale bar, 250 μm. **(D)** Quantitative analysis of tube formation assay. **(E)** Quantitative analysis of wounding migration assay. **(F–H)** The mRNA expression levels of bFGF, MMP-2 and MMP-9. *p < 0.05 as denoted by bar, **p < 0.01 as denoted by bar.

Cell proliferation assays, tube formation assays and wound migration assays were performed to assess the effects of betaine on angiogenesis *in vitro*. The results showed that HUVEC proliferation was significantly suppressed by betaine treatment (Figure 5A). Migration and morphological differentiation of endothelial cells into capillary-like structures are important steps during angiogenesis. Extensive capillary-like network formation and migration of endothelial cells were dramatically inhibited by betaine compared with the vehicle-treated group (Figures 5B–E). Betaine administration also decreased the expression level of angiogenic factors, including basic fibroblast growth factor (bFGF) (Figure 5F). Extracellular proteolytic activity regulation through the basement membrane is an important step in endothelial cell migration. Similarly, the expression levels of MMP 2 and MMP 9, two major molecules involved in the degradation of the extracellular membrane, were markedly decreased by betaine treatment (Figures 5G,H). These data suggest that betaine could suppress blood vessel formation *in vitro*.

### Betaine Inhibits Aberrant Vessel Formation in OA Mice

**FIGURE 6:**
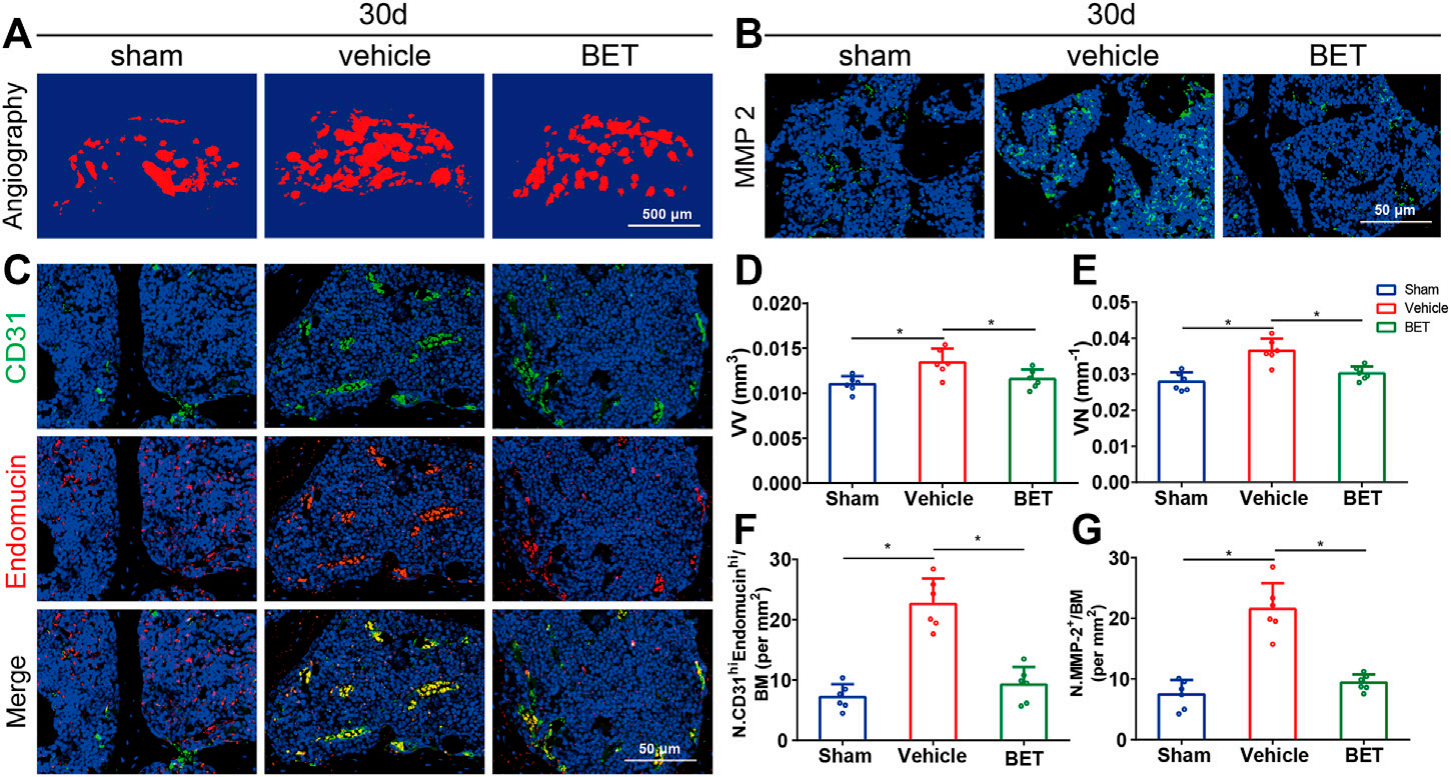
Betaine inhibits aberrant vessel formation in ACLT mice. **(A)** 3D microangiography of medial compartment of tibial subchondral bone 30 days post operation. Scale bar, 500 μm. **(B)** Representative immunofluorescence staining of MMP-2 in ACLT mice 30 days after operation. Scale bar, 50 μm. **(C)** Representative double staining of CD31 positive and Endomucin positive cells 30 days post operation. Scale bar, 50 μm. **(D)** Quantitative analysis of vessel volume relative to tissue volume (VV/TV). **(E)** Quantitative analysis of vessel number (VN). **(F)** Quantification of CD31 positive and Endomucin positive cells 30 days post operation. **(G)** Quantitative analysis of MMP-2 positive cells 1-month post operation. *p < 0.05 as denoted by bar, **p < 0.01 as denoted by bar.

Microangiography was applied to explore the possible influence of betaine on subchondral bone vessel formation. The results showed that the number and volume of microvasculature in subchondral bone marrow were substantially increased in vehicle-treated group mice at 30 d. However, betaine inhibited the advancement of angiogenesis and retained blood vessel formation identical to sham controls (Figures 6A,D,E). CD31 and endomucin double immunofluorescence staining was adopted to identify the type of blood vessels suppressed by betaine treatment. The results indicated that CD31^hi^Emcn^hi^ vessel formation was considerably increased in the subchondral bone marrow of the vehicle-treated groups at 30 d, while changes in blood vessels were abrogated by betaine treatment (Figures 6C,F). Consistently, MMP-2 levels were significantly upregulated in mice in the vehicle-treated group at 30 d, whereas betaine administration reduced MMP-2 expression to a level similar to that of sham controls (Figures 6B,G). Taken together, these results indicate that betaine could inhibit aberrant vessel formation in ACLT mice.

### Betaine Inhibits RANKL-Induced Reactive Oxygen Species Production and the MAPK Signaling Pathway.

**FIGURE 7:**
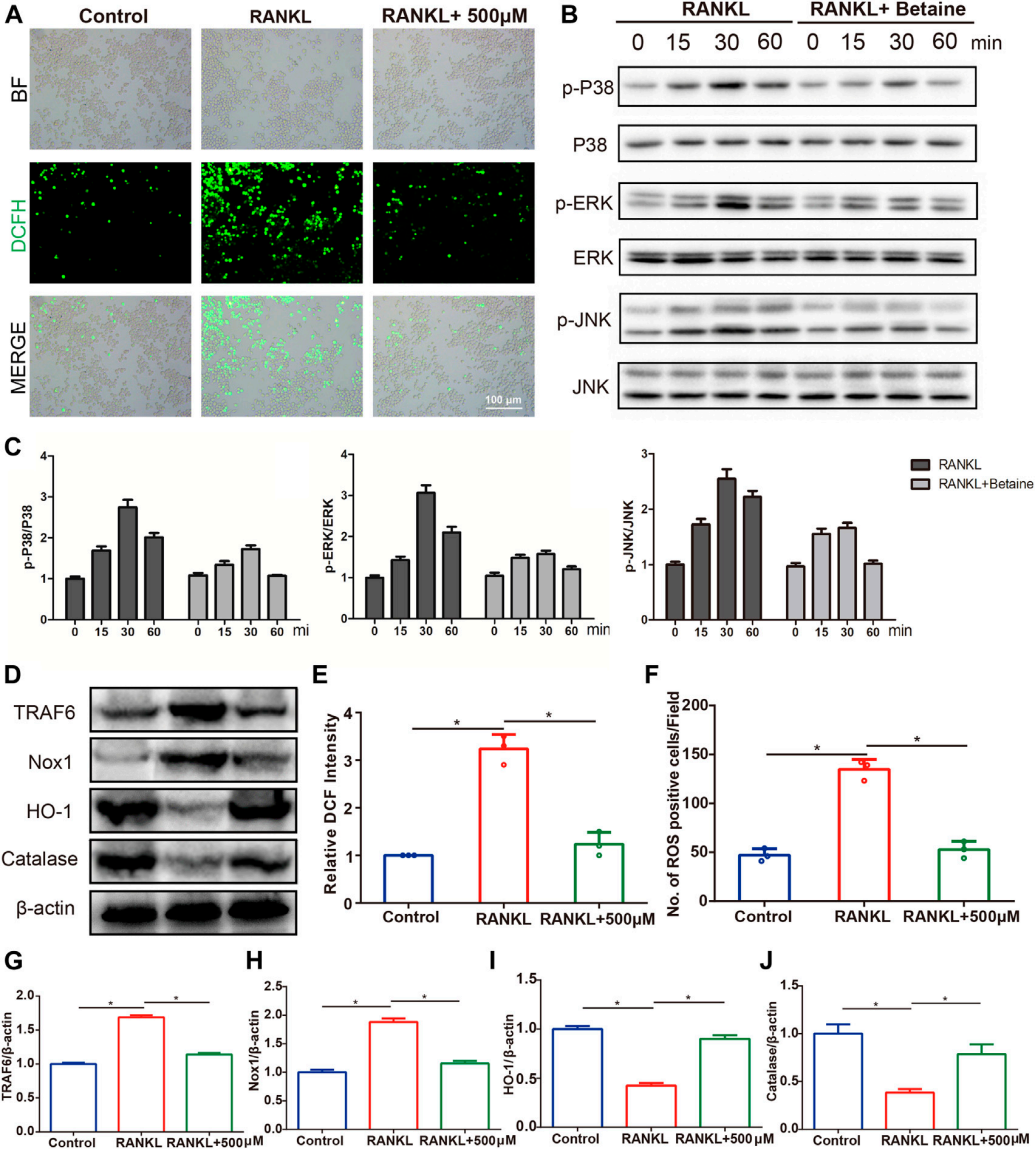
Betaine inhibits RANKL-induced activation of reactive oxygen species (ROS) and MAPK signaling pathway. **(A)** Representative images of ROS production in BMMCs with or without betaine treatment. BF, bright field. DCFH-DA, 2′,7′-dichlorofluorescin diacetate. Scale bar, 100 μm. **(B,C)** Western blot analysis of the phosphorylation of ERK, P38, and JNK in different groups and time points. **(D)** Western blot analysis of the expression levels of TRAF6, Nox1, HO-1 and Catalase in different groups. **(E,F)** Quantitative analysis of relative DCF intensity and the number of ROS positive cells per field. **(G–J)** Quantification of the expression levels of TRAF6, Nox1, HO-1 and Catalase. *p < 0.05 as denoted by bar.

DCFH-DA staining was conducted to investigate whether betaine administration inhibited osteoclastogenesis by regulating ROS production. The results showed that the intensity of DCF fluorescence and ROS-positive cells were considerably upregulated in RANKL-induced BMMCs compared with control group cells. However, betaine treatment significantly decreased these indexes (Figures 7A,E,F). Moreover, the expression levels of TRAF6 and Nox1, two major contributing molecules in ROS generation, were upregulated by M-CSF and RANKL induction but were markedly inhibited by betaine treatment (Figures 7D,G,H). The expression of antioxidant enzymes, including HO-1 and catalase, was significantly enhanced by betaine treatment (Figures 7D,I,J). To further investigate the mechanism of betaine treatment on osteoclastogenesis, we characterized the phosphorylation levels of three major subfamilies of MAPK signaling, P38, ERK and JNK. The results showed that the phosphorylation levels of the three major subfamilies were significantly increased after M-CSF and RANKL induction and were substantially inhibited by betaine treatment (Figures 7B,C). Overall, these results demonstrate that betaine suppresses osteoclastogenesis by reducing ROS production and inhibiting MAPK signaling activation.

## Discussion

In this study, we found that betaine preserved the subchondral bone architecture and abrogated aberrant blood vessel formation to prevent articular cartilage degeneration and attenuate OA progression *in vivo*. In addition, we showed that betaine suppressed osteoclastogenesis both in the subchondral bone marrow and *in vitro*. Mechanistically, betaine inhibited RANKL-induced ROS production and the MAPK pathway.

Chromatography is a biological affinity chromatographic technology that shows great latent capacity in screening possible active constituents from complex systems (Ding et al., 2014; Gu et al., 2019; Gu et al., 2020). Many studies have investigated the interactions between the cell membrane and possible active drugs. Compounds with better retention behavior were expected to have stronger bonds with cell membrane receptors (Chen et al., 2014; Ahmad Dar et al., 2020). We previously identified several active components by using the BMMC/CMC analytical system (Gu et al., 2020; Li et al., 2020). In this study, BMMCs were used as target cells for screening potential compounds from the Lycii Radicis Cortex. One component with better retention behavior on BMMCs was screened and identified as betaine by comparison with the LRC chemical composition database. We first confirmed that betaine could inhibit osteoclast formation and bone resorption function *in vitro*.

Osteoarthritis is considered a whole-joint disease characterized by articular cartilage deterioration, subchondral bone edema and sclerosis, abnormal angiogenesis and osteophyte formation (Kapoor et al., 2011; Goldring, 2012). Biomechanical and biochemical interactions between the underlying subchondral bone and overlying articular cartilage influence the hemostasis and integrity of cartilage (Sharma et al., 2013). The integrity and stability of subchondral bone provides a physiological environment and mechanical support for articular cartilage of the joint. Subchondral bone undergoes constant remodeling progression, including bone resorption and formation regulated by osteoclasts and osteoblasts, while osteoclasts play a pivotal role in this process (Castañeda et al., 2012; Li et al., 2013). Increased osteoclast-mediated bone resorption was observed in patients with progressive knee osteoarthritis compared with those with nonprogressive disease (Bettica et al., 2002). Several studies have also suggested that osteoclast activity and bone resorption in subchondral bone are significantly increased in the early phase of OA progression as a consequence of mechanical loading instability of joints (Karsdal et al., 2014). In particular, as early as 7 days after ACLT operation, the number of osteoclasts in subchondral bone was significantly upregulated before articular cartilage degradation became evident (Zhen et al., 2013). Subchondral bone remodeling plays a crucial role during OA development, especially in the early stage (Pan et al., 2009; Roemer et al., 2009; Tanamas et al., 2010). Subchondral bone microarchitecture was maintained by normal osteoclast activity and coupled bone remodeling. In the early phase of OA progression, the over-activated osteoclasts lead to a substantial increase in bone resorption and uncoupled bone remodeling, which ultimately results in OA onset (Hayami et al., 2012; Khorasani et al., 2015). Inhibition of osteoclast activity and bone resorption by bisphosphate prevents cartilage loss and reduces OA progression and pain (Bingham et al., 2006; Kadri et al., 2010; Strassle et al., 2010). As betaine was demonstrated to have inhibitory effects on osteoclastogenesis *in vitro*, we focused on the possible impact of betaine on ACLT-induced OA mice.

In our study, we observed that TRAP^+^ osteoclast cells and bone resorption levels in subchondral bone were significantly increased after ACLT at 14 d, while betaine treatment significantly inhibited osteoclastogenesis and maintained the microarchitecture of subchondral bone. Cartilage preservation is the most important problem in the treatment of OA (Findlay and Kuliwaba, 2016). We then examined the effects of betaine on articular cartilage preservation and observed that betaine significantly prevented articular cartilage degeneration and decreased OARSI scores relative to ACLT mice at 30 and 60 d. Collagen X, a hypertrophic chondrocyte marker, and MMP-13, a cartilage-damage collagenase, were significantly increased, while lubricin, a cartilage-protection marker and hyaline cartilage thickness, was significantly decreased after surgery at 30 d. These findings are consistent with several previous studies (Cui et al., 2016; Mu et al., 2018). However, the values of these parameters were significantly normalized by betaine administration. These results imply that betaine preserves the integrity and homeostasis of articular cartilage by inhibiting overactivated osteoclast activity in subchondral bone.

Angiogenesis and adequate blood supply are critical to bone formation (Portal-Núñez et al., 2012; Percival and Richtsmeier, 2013). Neoangiogenesis and microcracks caused by excessive mechanical loading contribute to articular cartilage damage. High turnover of subchondral bone is accompanied by increased bone formation, which leads to subchondral bone sclerosis and final OA progression (Arnoldi et al., 1972; Lotz, 2012; Cui et al., 2015). TNF-α-induced LRG1 promotes angiogenesis, and the positive feedback of chondrocytes forming H-type blood vessels of subchondral bone promotes OA progression in mice (Wang et al., 2017; Lu et al., 2018). Targeting angiogenesis also reduces osteoarthritis progression and alleviates OA pain (Mapp and Walsh, 2012; Hamilton et al., 2016). Betaine has been reported to have inhibitory effects on aberrant angiogenesis (Yi and Kim, 2012; Park et al., 2017). In this study, we observed that microvasculature formation in subchondral bone marrow was significantly increased in OA mice at 30 d. We further demonstrated that CD31^hi^Emcn^hi^ H-type vessels, which could couple angiogenesis with osteogenesis, were also significantly increased in OA mice at 30 d, while betaine treatment abrogated abnormal angiogenesis in subchondral bone. In this study, we demonstrated that Betaine suppressed vessel formation by both indirect manner and direct manners. For the indirect manner, betaine suppressed osteoclasts formation *in vitro* and *in vivo* as demonstrated, which is closely related to vessel formation in subchondral bone during osteoporosis progression. And Betaine could suppress the expression of the vessel formation related enzymes. MMP-2 produced by endothelial cells is a membrane-associated neutral endopeptidase that can promote angiogenesis by regulating the interactions between cells and the extracellular matrix. Betaine treatment showed an antifibrotic effect by diminishing MMP-2 expression levels in alcoholic liver fibrosis rats (Bingül et al., 2016). In our study, we found that MMP-2 expression was dramatically increased in subchondral bone after ACLT at 30 d, whereas betaine treatment significantly decreased MMP-2 levels equivalent to sham controls. We also demonstrated that betaine could suppress angiogenesis *in vitro* by inhibiting bFGF, MMP 2 and MMP 9 expression. These results show that betaine can reduce the progression of OA by inhibiting angiogenesis in subchondral bone.

Osteoclasts are bone-specialized multinucleated cells produced by the differentiation of macrophage-derived hemopoietic progenitors induced by RANKL (Asagiri and Takayanagi, 2007). RANKL binds with its receptors on the cell membrane to increase ROS production *via* a production signaling cascade including TRAF6 and Nox1 (Lee et al., 2005). The maintenance of ROS levels depends on the balance between the rate of generation and the rate of scavenging involving enzymes such as heme oxygenase-1 (HO-1) and catalase (Kanzaki et al., 2013). Increased ROS production acts as a second messenger and oxidizes tyrosine phosphatases, thus activating the subsequent MAPK pathway (Davis, 2000; Lee et al., 2005; Kanzaki et al., 2017). ROS and MAPK serve as two crucial mediators in RANKL-induced osteoclast differentiation progression, and inhibition of ROS production and MAPK signaling restrain osteoclastogenesis (Lee et al., 2005; Yip et al., 2005; Agidigbi and Kim, 2019; Chen et al., 2019; Ni et al., 2020). Betaine has been reported to inhibit ROS and MAPK in previous studies (Kim et al., 2014; Park et al., 2017; Jiang et al., 2019; Veskovic et al., 2019). In our study, we found that betaine significantly suppressed ROS levels by inhibiting ROS production and upregulating ROS scavenging. We also observed that betaine inhibits P38, ERK and JNK activation in BMMCs induced by RANKL, indicating that betaine suppresses osteoclastogenesis by inhibiting the ROS and MAPK pathways.

The inflammatory system has also been reported to be involved in the progression of OA (Kapoor et al., 2011; Herrero-Beaumont et al., 2019). Betaine was shown to suppress lipopolysaccharide (LPS)-induced inflammation-related cytokines in RAW 264.7 cells in an *in vitro* study (Kim et al., 2014). Another study found that betaine treatment inhibited the Toll-like receptor 4 (TLR-4)/NF-κB pathways to restore fructose-induced astrogliosis and inflammation (Li et al., 2015). Betaine also has inhibitory effects on interleukin (IL)-1β production and inflammasome activation (Fan et al., 2014; Ge et al., 2016; Xia et al., 2018). These may indicate an additional mechanism underlying the action of betaine in OA progression. Further study is still needed to elucidate the mechanisms and roles of betaine in the treatment of OA and other diseases.

Overall, we have shown for the first time that betaine attenuates OA progression in ACLT models by targeting subchondral bone pathological features. Betaine could prevent subchondral bone microarchitecture changes, inhibit hyperactivated osteoclast-induced bone resorption, and reduce aberrant microangiogenesis. Our findings expanded the possible clinical description of betaine and suggest that betaine could be an effective candidate for OA therapy.

## Data Availability

The raw data supporting the conclusions of this article will be made available by the authors, without undue reservation.
